# Anatomical and functional correlation in Susac syndrome: multimodal imaging assessment

**DOI:** 10.1186/s40942-017-0092-9

**Published:** 2017-10-16

**Authors:** Alexandre G. B. Azevedo, Luiz H. Lima, Léo Müller, Flávio Rezende Filho, Cláudio Zett, André Maia, Luiz Roisman

**Affiliations:** 1 Departamento de Oftalmologia – Secretaria Administrativa, Universidade Federal de São Paulo, Rua Botucatu, 821, 1o Andar, São Paulo, 04023-062 Brazil; 2 Department of Neurology, Federal University of São Paulo, São Paulo, Brazil; 3 Carrera de Tecnología Médica, Facultad de Ciencias, Pontificia Universidad Católica de Valparaíso, Valparaiso, Chile

**Keywords:** Microperimetry, Optical coherence tomography angiography, Retinal artery occlusion, Susac syndrome, Visual field testing

## Abstract

**Background:**

Susac’s syndrome (SuS) is an uncommon disease characterized by retinal microangiopathy that may be assessed more accurately with optical coherence tomography angiography (OCTA), a new imaging technique which provides a retinal microvasculature map. The purpose of this case report is to describe the multimodal imaging findings of SuS correlating OCTA with functional tests.

**Case presentation:**

Retrospective review of one case with clinical and imaging evidence of SuS. Color fundus photograph, fluorescein angiography (FA), OCTA, microperimetry (MP) and visual field (VF) tests were analyzed at the time of presentation and at 1- and 6-month visit following initiation of treatment. The study patient underwent standard treatment for SuS. The patient age was 31 year-old and the baseline visual acuity was 20/60 and 20/20 in the right and left eyes, respectively. At presentation, FA showed branch retinal arterial occlusion within the macular area of the right eye and vascular leakage in the periphery of the left eye. OCTA demonstrated areas of superficial and deep retinal vascular plexuses hypoperfusion in both eyes. The OCTA segmentations in the outer retina and choriocapillaris were normal. The low VF and MP sensitivity signals precisely corresponded to the topography of decreased vascular perfusion seen on the OCTA density map in both eyes. Six months after specific SuS therapy, retinal vascular perfusion showed partial improvement in both eyes.

**Conclusion:**

OCTA may demonstrate superficial and deep retinal vascular non-perfusion without choriocapillary vasculature changes in SuS. This anatomical information given by OCTA corresponded to points of low sensitivity on functional tests represented by VF and MP.

Susac’s syndrome (SuS) is an uncommon autoimmune disease characterized by a microangiopathy that typically involve the brain, retina and inner ear leading to the clinical triad of encephalopathy, branch retinal arterial occlusion (BRAO), and neuro-sensorial deafness. SuS occurs more often in females between the third and fourth decades of life, and the microvascular injury by anti-endothelial cell antibodies is the most accepted disease pathogenesis [1, 2].

The diagnosis of SuS may be challenging since its signs and symptoms may not appear simultaneously and other clinical entities may overlay the disease clinical features [3–5]. Multimodal imaging and multidisciplinary approach are frequently used to confirm SuS diagnosis [6, 7]. Brain magnetic resonance imaging (MRI) characteristically depicts corpus callosum and periventricular white matter lesions and vascular micro-infarctions [8, 9]. Pure tone audiometry commonly reveals bilateral neuro-sensorial hearing loss that typically affects low to mid hearing frequencies [10]. Retinal branch artery occlusion, vascular leakage and Gass plaques (arteriolar wall hyperfluorescence) represent the main ocular findings and are usually seen on fluorescein angiography (FA) [11]. Optical coherence tomography (OCT) may also help to differentiate SuS from other retinal vascular diseases because of the characteristic morphological pattern of injury to the inner retina [12, 13].

Optical coherence tomography angiography (OCTA) is a novel noninvasive imaging technique that provides volumetric retinal and choroidal blood flow data. The registration of erythrocyte movement with repeated scans allows the development of a retinal microvasculature map that may assess retinal vascular diseases more accurately [14]. The purpose of this case report is to describe the OCTA findings in a patient with SuS and correlate with functional tests, such as microperimetry (MP) and visual field (VF).

## Case report

**Fig. 1:**
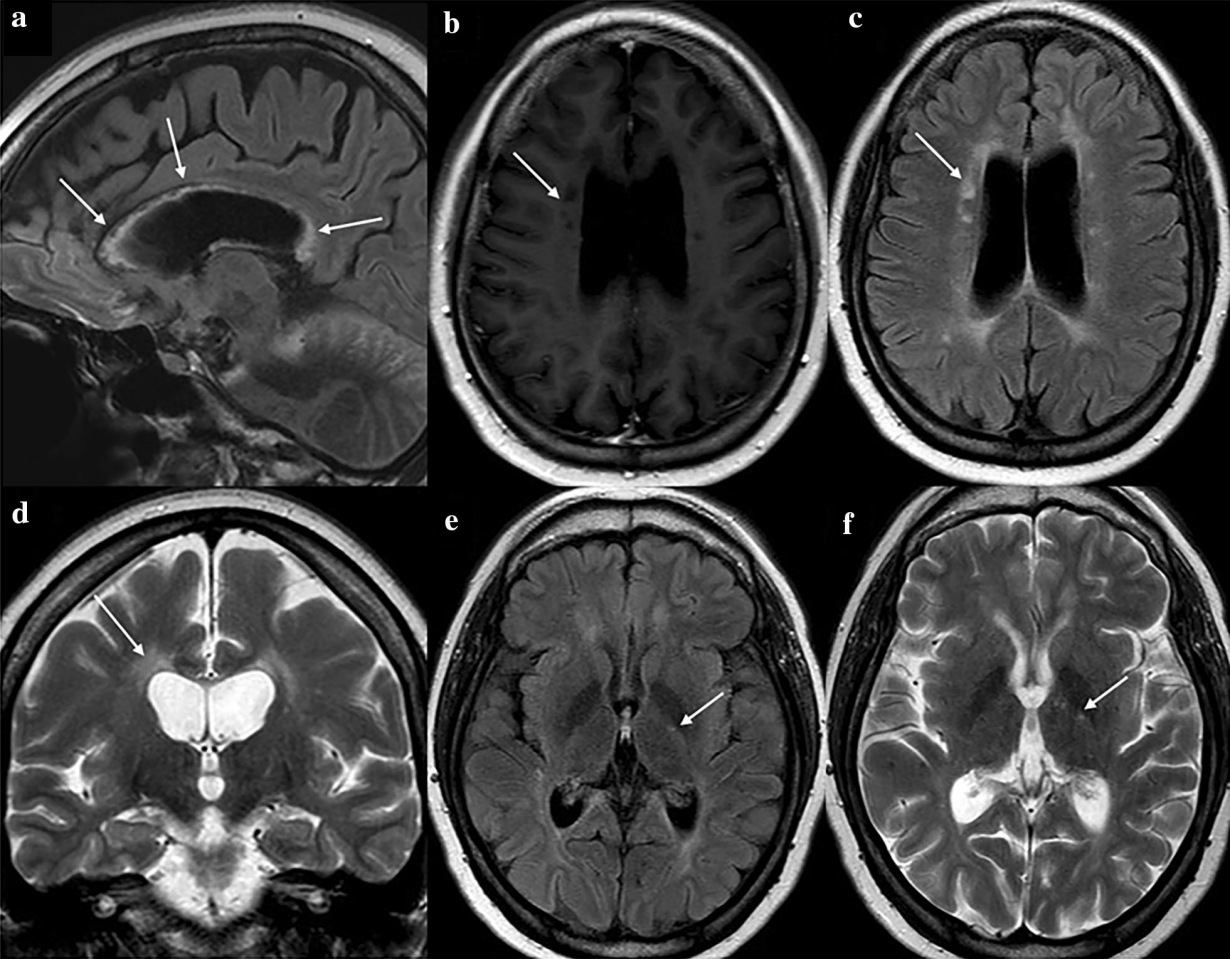
Brain magnetic resonance imaging (MRI). **a** Sagittal FLAIR-weighted sequence demonstrated diffuse hyperintense signal in the corpus callosum, **b** axial T1-weighted sequence showed multiple periventricular black holes, **c**, **d** axial FLAIR-weighted and coronal T2-weighted sequences showed periventricular lesions, **e**, **f** axial FLAIR-weighted and axial T2-weighted sequences demonstrated small lesions with hyperintense signal in the internal capsule

A 31 year-old woman presented to clinic complaining of visual loss in the right eye and hearing loss. She had a past medical history of Hashimoto’s thyroiditis and was taking levothyroxine 75 mg/day at the time of first visit. The cerebrospinal fluid exam showed lymphocytic pleocytosis (88 cells/mm^3^ and a total protein of 78 mg/dl) without oligoclonal bands. Brain MRI demonstrated hyperintense signal in the corpus callosum and multiple periventricular black holes which are respectively consistent with corpus callosum thinning and periventricular white mass microinfarctions (Fig. 1).

**Fig. 2:**
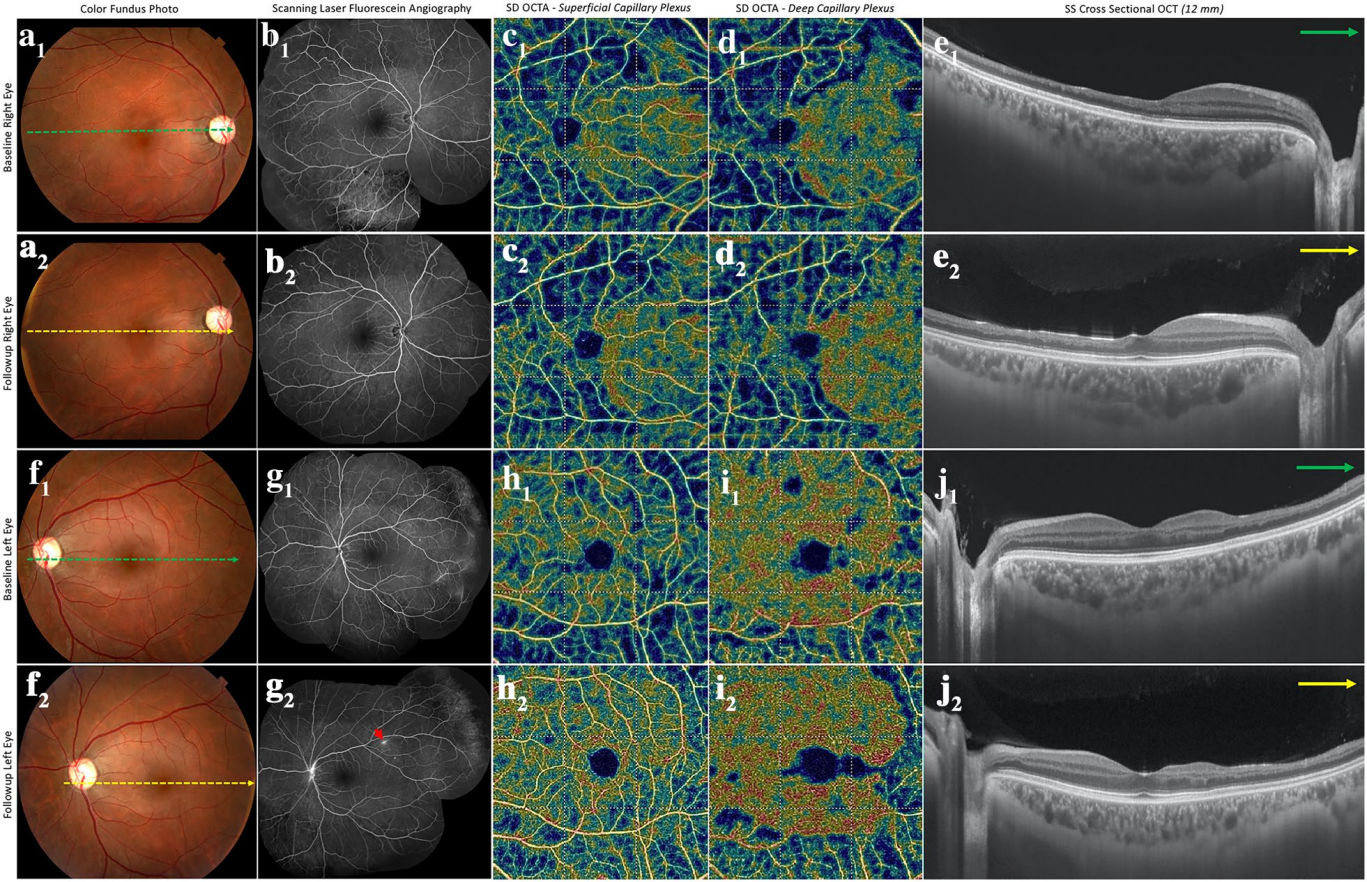
**a1**, **b1**, **c1**, **d1**, **e1** Color fundus photograph (**a1**) of the right eye showed a superior temporal arteriolar narrowing within macular area. Fluorescein angiography (FA) (**b1**) depicted a branch retinal arterial occlusion (BRAO) in the superior temporal macula of the right eye. Optical coherence tomography angiography (OCTA) perfusion map 6 × 6 mm of superficial and deep capillary plexuses (**c1**, **d1**) of the right eye revealed temporal parafoveal hypoperfusion. B-scan OCT (**e1**) of the right eye showed thinning of the inner retina temporal to the fovea, **f1**, **g1**, **h1**, **i1**, **j1** color fundus photograph (**f1)** of the left eye showed no abnormalities within the macula area. FA (**g1**) demonstrated non-perfusion associated with leakage in the temporal periphery of the left eye. OCTA perfusion map 6 × 6 mm of superficial and deep capillary plexuses (**h1**, **i1**) of the left eye revealed small spots of decreased vascular perfusion within the macular area. Thinning of the inner retina temporal to the fovea was also observed on B-scan OCT in the left eye (**j1**), **a2**, **b2**, **c2**, **d2**, **e2** at 6-month follow-up, although color fundus photograph (**a2**) of the right eye did not reveal any change in comparison with the baseline exam, the signs of BRAO disappeared on FA (**b2**). OCTA perfusion map 6 × 6 mm of superficial and deep capillary plexuses (**c2**, **d2**) of the right eye showed an impressive improvement of vascular perfusion in the previous baseline hypoperfused areas, **e2** thinning of the inner retina temporal to the fovea of the right eye remained at 6-month follow-up, **f2**, **g2**, **h2**, **i2**, **j2** at 6-month follow-up, the color fundus photograph (**f2**) of the left eye did not reveal any change in comparison with the baseline exam, and the FA (**g2**) revealed new vascular staining on the temporal superior vascular arcade (arrow) and maintenance of peripheral vascular leakage. OCTA perfusion map 6 × 6 mm of superficial and deep capillary plexuses (**h2**, **i2**) of the left eye showed improvement of previous small hypoperfused areas, **j2** thinning of the inner retina temporal to the fovea of the left eye remained at 6-month follow-up

On ocular examination, the best-corrected visual acuity (BCVA) was 20/60 in the right eye and 20/20 in the left eye. There were no anterior segment abnormalities, anterior chamber reaction, vitritis or impaired pupillary light reflex in both eyes. Color fundus photograph (TRC50DX, Topcon, Japan) of the right eye revealed a superior temporal arteriolar narrowing within macular area and was unremarkable in the left eye (Fig. 2a1, f3). FA (HRA, Heidelberg Engineering, Germany) showed BRAO in the superior temporal macula of the right eye and vascular leakage in the temporal peripheral retina of the left eye (Fig. 2b1, g3). B-Scan OCT (Spectral-domain OCT Spectralis, Heidelberg Engineering, Germany and DRI Swept Source OCT Triton, Topcon, Japan) demonstrated inner retinal thinning on the temporal side of the macula in both eyes (Fig. 2e1, j3). This temporal retinal thinning was more pronounced in the right eye. OCTA (Spectral-domain OCT RTVue-XR Avanti, Optovue, United States and DRI Swept Source OCT Triton, Topcon, Japan) revealed vascular hypoperfusion within the macular area in both superficial and deep capillary retinal plexuses (Fig. 2c1–d1, h3–i3). The area of OCTA vascular hypoperfusion corresponded to the topography of BRAO seen on FA in the right eye. The OCTA segmentations in the outer retina and choriocapillaris were unremarkable in both eyes.

**Fig. 3:**
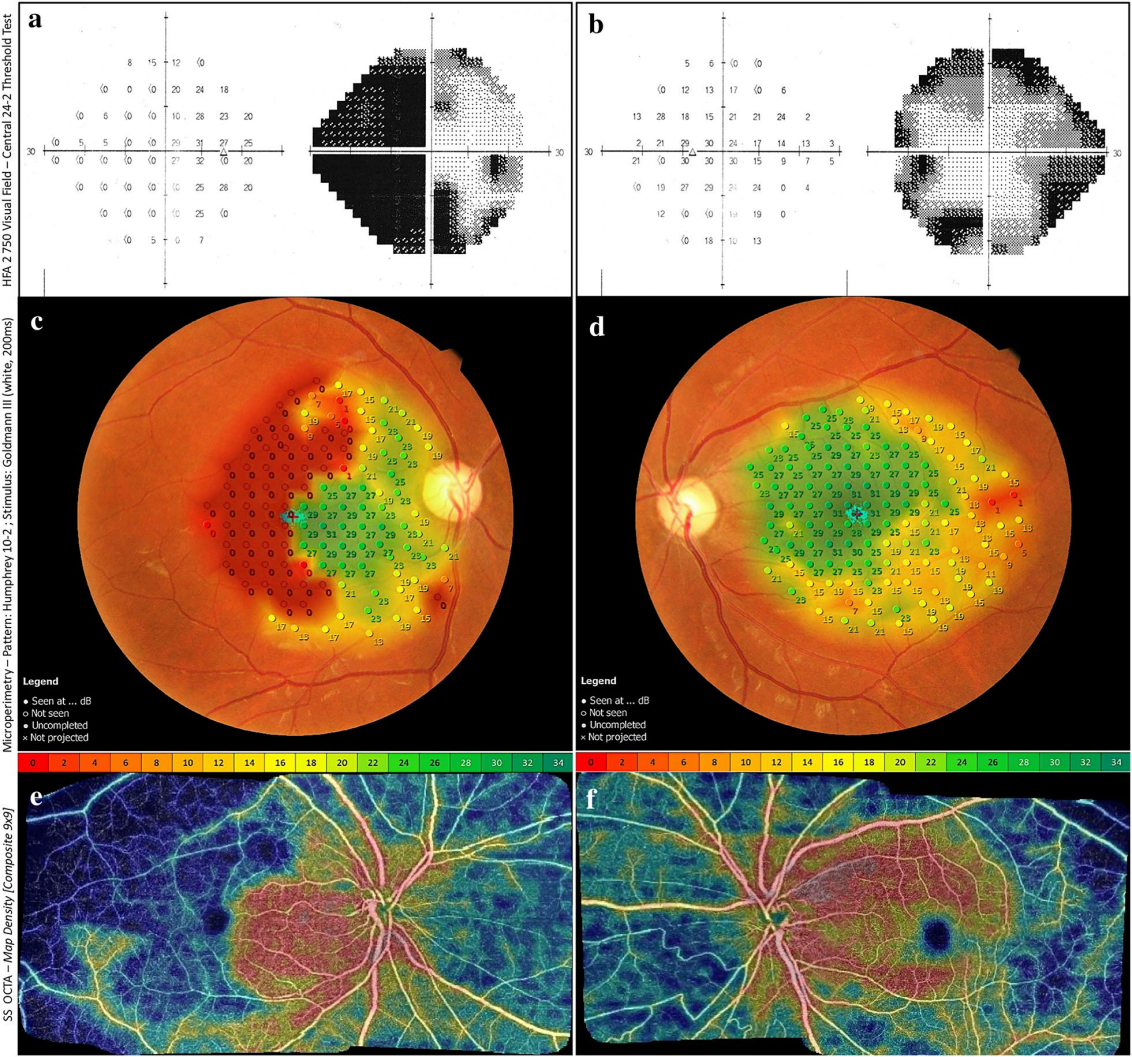
**a** Visual field test (24-2 strategy) of the right eye revealed nasal, central and paracentral temporal sensitivity loss, **b** visual field test (24-2 strategy) of the left eye showed visual field loss in the nasal periphery, sparing the central vision, **c**, **e** microperimetry of the right eye showed a large area of low sensitivity in the temporal macula that corresponded to the same topography of decreased vascular perfusion on OCTA 9 × 9 mm composite image of the superficial capillary plexus perfusion map, **d**, **f** microperimetry of the left eye showed a very small area of low sensitivity temporally to the macula that corresponded to the same topography of decreased vascular perfusion on OCTA 9 × 9 mm composite image of the superficial capillary plexus perfusion map

The functional tests VF (SITA strategy, stimulus III White, Humphrey HFA II 750, Carl Zeiss Meditec, Germany) and MP (MP-3, Nidek Technologies, Italy) showed visual function loss in both eyes (Fig. 3a–d). VF demonstrated nasal, central and paracentral decreased sensitivity in the right eye, and solely peripheral nasal decreased sensitivity in the left eye. MP depicted decreased sensitivity in the upper, inferior and temporal macula in the right eye, and decreased sensitivity in the temporal macula in the left eye. These low VF and MP sensitivity signals corresponded to the decreased vascular perfusion seen on the OCTA density map (Fig. 3e, f).

The patient was treated with methylprednisolone pulse therapy intravenously 1000 mg/day for 3 days, and the BCVA improved to 20/40 in the right eye at 1-month follow-up. A decrease in the left eye visual acuity (from 20/20 to 20/40) was noted at 6-month follow-up. Although fundus color photograph (Fig. 2a2, f4) did not reveal any change in comparison with the baseline fundus exam (Fig. 2a1, f3), a vascular leakage was observed in the retinal periphery of the left eye (Fig. 2b2, g4). In the right eye, at baseline, the vascular density index was 38.85 and 36.61% in the superficial and deep capillary plexuses, respectively. At 6-month follow up visit it was 40.14 and 41.78% in superficial and deep capillary plexuses respectively. In the left eye, at baseline, the vascular density index was 40.71 and 50.15% in the superficial and deep capillary plexuses, respectively. At 6-month follow up visit, it was 47.67% in superficial and 50.7% in the superficial and deep capillary plexuses, respectively. (Fig. 2c2–d2, h4–i4).

A new course of methylprednisolone pulse therapy followed by oral prednisone (20 mg q24 h) and azathioprine (50 mg q12 h) was initiated. Following this treatment, the patient became asymptomatic and the BCVA returned to 20/20 in both eyes.

## Discussion

SuS is a rare condition characterized by microvessel occlusions that lead to hypoxic retinal lesion [1, 2]. As SuS typically affects the retinal microvasculature without choroidal vascular damage, the inner retinal layers thinning is a common disease consequence. Conversely, the outer nuclear and photoreceptor layers are not affected as they are predominantly supplied by choroidal vasculature. These pathologic changes are often observed in the temporal macular and a loss of foveal delineation may occur as a consequence of retinal thinning due to the chronic hypoxic damage [12, 13]. Besides the clinical imaging, laboratory and imaging exams consistent with SuS diagnosis, our case also presented with bilateral atrophy and hypoxic damage in the temporal and superior quadrants. The central macula remained preserved with normal central macular thickness and mild visual acuity deficit as it is usual in SuS patients.

OCTA is a new diagnostic tool, especially for diseases that cause retinal vascular lesion or neovascularization, allowing a noninvasive assessment and follow-up of retinal tissue damage over time. In agreement with the current OCTA literature on SuS [15], the present case demonstrated an unaffected choriocapillaris vasculature and both superficial and deep retinal vascular plexuses damage characterized by vascular non-perfusion. This vascular injury was more precisely demonstrated by OCTA than the FA. Interestingly, the superficial capillary plexus had a more pronounced drop in the vascular density in the right eye, but the deep capillary plexus was more affected in the left eye. If we consider as clinically significant an increase in vascular density of 10% or more, the superficial capillary plexus in the right eye and the deep capillary plexus in the left eye showed a positive variation of vascular flow measured by the vascular density index on the perfusion map. At 6-month follow-up, the vascular density in both superficial and deep capillary plexus showed an improvement in both eyes. We hypothesized that this variation could be explained by a vascular flow change related to disease activity. The variation of vascular flow on the perfusion map with vascular density index that was noted on the superficial capillary plexus of the left eye could be explained by a vascular flow change related to disease activity. Differently from a complete retinal arterial occlusion, a retinal vasculitis could lead to a lower vascular flow because of the vessel narrowing. This lower vascular flow usually increases following the disease control. Although this information might give an indirect clue of disease activity, we must be aware of a natural oscillation of the vascular flow and presence of several artifacts that can affect the OCTA interpretation [16].

Although OCTA may provide high quality images, these images are limited to the posterior pole, and the confirmation of disease activity in the retinal periphery is still dependent on FA imaging. In our case, the OCTA identified both the injured vascular area and capillary dropout more precisely than the FA. Although OCTA can not demonstrate signs of disease activity since it is not able to detect vascular leakage or vessel staining, it may allow a more accurate anatomical-functional correlation with MP and VF when analyzing the retinal non-perfused areas. These low VF and MP sensitivity signals observed in both eyes of this present case corresponded to the decreased perfusion seen on the OCTA density map. In spite of segmental VF defects had been reported in association with involved arterioles in SuS [17–19], the current case is the first to describe such association with OCTA.

In conclusion, OCTA may provide a more detailed assessment of microvascular changes, allowing a more precise anatomical-functional correlation in SuS. Despite the fact that there is another case report [15] in the literature describing OCTA findings in SuS, to the best of our knowledge, the present case is the first to address the SuS vascular changes using the vascular density map, and to correlate the SuS microvascular changes with functional ocular tests such as MP and VF. This report has some limitations which have to be pointed out, such as the singular patient reported and the limited patient follow-up. Larger series with long-term follow-up of SuS cases imaged with OCTA are needed for a better retinal microvascular changes understanding in this disease.

## Abbreviations

BCVA: best-corrected visual acuity
BRAO: branch retinal arterial occlusion
FA: fluorescein angiography
MP: microperimetry
MRI: magnetic resonance imaging
OCT: optical coherence tomography
OCTA: optical coherence tomography angiography
SuS: Susac’s syndrome
VF: visual field
